# Supplementary material for: Distinct Immunophenotypes in the DNA Index-Based Stratification of Pediatric B-Cell Acute Lymphoblastic Leukemia
Source: Cancers (Basel). 2024 Oct 24;16(21):3585. doi: 10.3390/cancers16213585 (PMC11545691; doi:10.3390/cancers16213585)
Supplement: Supplementary file 1 [file cancers-16-03585-s001.zip › cancers-3226113-supplementary.pdf]

Supplementary Material

Supplementary Table S1. Antibody panels used in immunophenotyping of B-ALL patients.

B panel

Antibody	Fluorochrome	Clone	Manufacturer	Catalog number	Dilution	Isotype
CD5	Brilliant Violet 605	L17F12	BioLegend	364020	5 µl/ 1 × 10^6 cells in 100µl	IgG2 Mouse
CD10	Brilliant Violet 421	HI10a	BioLegend	312218	5 µl/ 1 × 10^6 cells in 100µl	IgG1 Mouse
CD19	APC	J3-119	Beckman Coulter	IM2470	5 µl/ 1 × 10^6 cells in 100µl	IgG1 Mouse
CD20	PC5	B9E9	Beckman Coulter	IM2644U	5 µl/ 1 × 10^6 cells in 100µl	IgG2 Mouse
CD22	APC/Cyanine7	HIB22	BioLegend	302528	5 µl/ 1 × 10^6 cells in 100µl	IgG1 Mouse
CD34	PE	Imm133	Beckman Coulter	IM1420	5 µl/ 1 × 10^6 cells in 100µl	IgG1 Mouse
CD38	Brilliant Violet 650	HB-7	BioLegend	356620	5 µl/ 1 × 10^6 cells in 100µl	IgG1 Mouse
CD45	KrO	J33	Beckman Coulter	B36294	5 µl/ 1 × 10^6 cells in 100µl	IgG1 Mouse
HLA-DR	APC-A750	Immu-357	Beckman Coulter	B42021	5 µl/ 1 × 10^6 cells in 100µl	IgG1 Mouse

T panel

Antibody	Fluorochrome	Clone	Manufacturer	Catalog number	Dilution	Isotype
CD2	PE-Cy5	RPA-2.10	Becton Dickinson	555328	20 µl/ 1 × 10^6 cells in 100µl	IgG1 Mouse
CD3	PC7	UCHT1	Beckman Coulter	6607100	5 µl/ 1 × 10^6 cells in 100µl	IgG1 Mouse
CD4	Brilliant Violet 650	SK3	Becton Dickinson	563875	5 µl/ 1 × 10^6 cells in 100µl	IgG1 Mouse
CD7	PE/Dazzle 594	Cd7-6b7	Biolegend	343120	5 µl/ 1 × 10^6 cells in 100µl	IgG2 Mouse
CD8	FITC	SK1	Becton Dickinson	347313	20 µl/ 1 × 10^6 cells in 100µl	IgG1 Mouse
CD34	PE	581	Beckman Coulter	IM1871U	20 µl/ 1 × 10^6 cells in 100µl	IgG1 Mouse
CD45	KrO	J33	Beckman Coulter	B36294	5 µl/ 1 × 10^6 cells in 100µl	IgG1 Mouse

Myeloid panel

Blanco	Fluorochrome	Clone	Manufacturer	Catalog number	Dilution	Isotype
CD11b	Brilliant Violet 605	ICRF44	Becton Dickinson	562723	5 µl/ 1 × 10^6 cells in 100µl	IgG1 Mouse
CD13	APC-R700	Wm15	Becton Dickinson	565124	5 µl/ 1 × 10^6 cells in 100µl	IgG1 Mouse
CD14	APC	RMO52	Beckman Coulter	IM2580U	10 µl/ 1 × 10^6 cells in 100µl	IgG2 Mouse
CD15	FITC	MMA	BioLegend	394706	5 µl/ 1 × 10^6 cells in 100µl	IgM Mouse
CD16	PC7	3G8	Beckman Coulter	6607118	5 µl/ 1 × 10^6 cells in 100µl	IgG1 Mouse
CD33	PE-CF594	CF594	Becton Dickinson	562492	5 µl/ 1 × 10^6 cells in 100µl	IgG1 Mouse
CD34	PE	581	Beckman Coulter	IM1871U	20 µl/ 1 × 10^6 cells in 100µl	IgG1 Mouse
CD45	KrO	J33	Beckman Coulter	B36294	5 µl/ 1 × 10^6 cells in 100µl	IgG1 Mouse
CD117	PE	104D2	Exbio	1P-586-T100	20 µl/ 1 × 10^6 cells in 100µl	IgG1 Mouse
HLA-DR	APC-A750	Immu-357	Beckman Coulter	B42021	5 µl/ 1 × 10^6 cells in 100µl	IgG1 Mouse

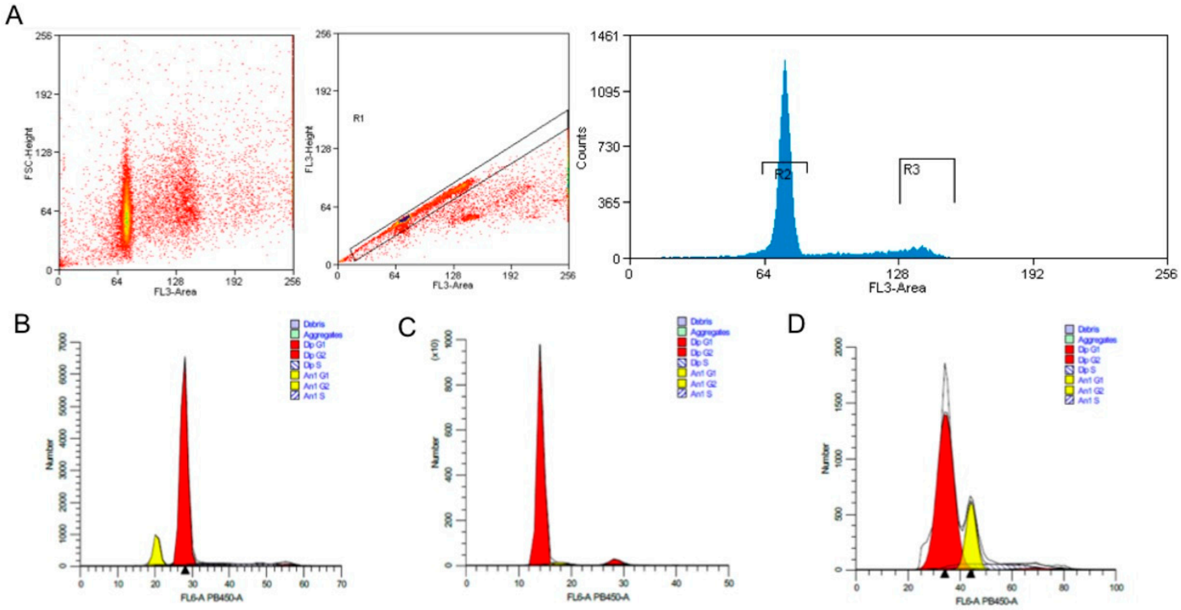

Figure S1: Analysis of DNA content and cell cycle phases using flow cytometry. (A) Representative gating strategy for selecting the cell population based on forward scatter (FSC) and side scatter (SSC) parameters, followed by FL3-Area and FL3-Height for doublet discrimination and subsequent DNA content analysis. (B-D) DNA content histograms showing the distribution of cells across different cell cycle phases (G0/G1, S, and G2/M phases) for hypoploid (B), normoploid (C), and hyperploid (D) B-ALL patients.
